# Long-Acting Antiretroviral Therapy for HIV via Drop-in Community-Based Care

**DOI:** 10.1001/jamanetworkopen.2026.20348

**Published:** 2026-06-26

**Authors:** Nicky J. Mehtani, Stephen J. Matzat, Kathleen B. O’Connor, Laura Cordoba, Sarah Strieff, Alix Strough, Morgan M. Philbin, Mallory O. Johnson, Monica Gandhi, Barry Zevin

**Affiliations:** 1 Whole Person Integrated Care, San Francisco Department of Public Health, San Francisco, California; 2 Department of Medicine, University of California, San Francisco, San Francisco

## Abstract

**Importance:**

People with HIV (PWH) experiencing homelessness face barriers to daily oral antiretroviral therapy (ART) adherence and often lack access to HIV specialty care. Data are needed on the feasibility and outcomes of long-acting ART (LA-ART) delivery within low-barrier, community-based settings.

**Objective:**

To evaluate the implementation and effectiveness of LA-ART delivery at a community-based drop-in clinic for people experiencing homelessness (PEH).

**Design, Setting, and Participants:**

This quality improvement study included PWH experiencing homelessness or housing instability, among whom substance use and psychiatric disorders were prevalent, receiving care at the Maria X. Martinez Health Resource Center, a municipal clinic serving PEH in San Francisco, California. Programmatic outcomes of LA-ART were evaluated from November 2021 through October 2025, with a secondary descriptive evaluation of PWH receiving standard treatment vs LA-ART between January 2023 and December 2024.

**Intervention:**

Intramuscular cabotegravir-rilpivirine injection, with or without lenacapavir, delivered within a high-touch model incorporating reminders, outreach, and flexible delivery compared with standard treatment consisting of oral ART when accepted.

**Main Outcomes and Measures:**

Outcomes were viral suppression (HIV RNA <200 copies/mL) and clinical encounters per person-year between 2023 and 2024. Among LA-ART recipients, additional outcomes included retention, injection timeliness, out-of-clinic administration, and virologic failure over the full program period.

**Results:**

Among 128 PWH included in the 2023-2024 descriptive cohort, 24 individuals (mean [SD] age, 44.6 [12.2] years; 15 cisgender males [63%]) received LA-ART, and 123 individuals (mean [SD] age, 42.6 [12.1] years; 69 cisgender males [66%]) received standard treatment (due to treatment transitions, treatment conditions were not mutually exclusive). All 24 (100%) individuals receiving LA-ART achieved and maintained viral suppression. Among those receiving standard treatment, 89 of 123 (72%) ever achieved viral suppression and 69 of 123 (56%) had viral suppression at last follow-up. The mean (SD) number of clinical encounters per person-year was higher for LA-ART recipients (23.0 [11.1] vs 9.1 [11.5]). Across the full program period, 30 individuals initiated LA-ART, and all continued receiving therapy through October 2025 (median duration [range], 106 [8-206] weeks). Of 637 injection visits, 43 (7%) were delayed by more than 7 days and 21 (3%) by more than 14 days, and 121 (19%) were administered outside the clinic. Three individuals experienced virologic failure while receiving cabotegravir-rilpivirine and achieved resuppression after the addition of lenacapavir.

**Conclusions and Relevance:**

In this quality improvement study, LA-ART delivery within a high-touch, flexible care model in a drop-in clinic serving PEH was associated with high rates of viral suppression. These findings highlight the potential for nonspecialty, community-based settings to deliver LA-ART to structurally vulnerable populations, while underscoring the substantial programmatic support required.

## Key Points

**Question:**

Can long-acting injectable antiretroviral therapy (LA-ART) for HIV be delivered in a community-based drop-in clinic for people experiencing homelessness, mental illness, and substance use disorders?

**Findings:**

In this quality improvement study of 128 people with HIV experiencing homelessness or housing instability over a 2-year period, all 24 (100%) receiving LA-ART achieved and maintained HIV viral suppression; among 123 prescribed standard treatment, 69 (56%) had viral suppression at last follow-up. LA-ART was delivered without requiring baseline viral suppression and involved frequent clinical encounters, reflecting a high-touch care model.

**Meaning:**

These findings indicate that when paired with intensive, flexible support, LA-ART can be delivered with high rates of viral suppression outside of traditional HIV specialty centers.

## Teaser

This quality improvement study examines the delivery of long-acting antiretroviral therapy for HIV in the flexible care setting of a drop-in clinic serving people experiencing homelessness.

## Introduction

People experiencing homelessness (PEH) in the US have more than 3 times the HIV prevalence of the general population and markedly lower rates of viral suppression.^1,2,3,4,5^ These disparities are driven in part by structural and psychosocial barriers to oral antiretroviral therapy (ART) adherence, including co-occurring substance use and psychiatric disorders, food insecurity, lack of safe medication storage, and frequent, often unpredictable disruptions in care continuity.^4,5,6^

Clinical trials have established the safety and efficacy of long-acting ART (LA-ART) for HIV treatment with cabotegravir-rilpivirine administered via intramuscular injection monthly or every other month.^7,8,9^ However, these pivotal studies enrolled only participants who had already achieved viral suppression with oral ART, largely excluding people with housing instability and active substance use. Demonstration projects at HIV specialty clinics, such as Ward 86 of Zuckerberg San Francisco General Hospital, suggest that LA-ART can be effective even among patients with viremia facing major challenges to oral ART adherence.^10,11,12^

These emerging data have informed updates to US Department of Health and Human Services and International Antiviral Society–USA guidelines, which now support cabotegravir-rilpivirine use in select individuals with HIV viremia and adherence barriers.^13,14,15^ However, most prior work has been conducted in HIV specialty settings, and little is known about LA-ART implementation in nonspecialty, community-based clinics serving PEH. This is particularly relevant following the recent US Food and Drug Administration approval of lenacapavir administered subcutaneously every 6 months for heavily treatment-experienced people with HIV (PWH).^16^

The Maria X. Martinez (MXM) Health Resource Center, part of San Francisco’s public health system, provides no-appointment, drop-in care for PEH, serving both PWH and those without HIV (ie, status neutral).^13^ By design, the MXM Health Resource Center and similar safety-net programs engage patients who have difficulty accessing conventional primary or specialty care due to unstable housing, substance use, and psychiatric illness, bridging essential medical services until more traditional clinical care can be established. Within this context, we conducted a quality improvement study to evaluate the integration of LA-ART at MXM Health Resource Center, reporting implementation and effectiveness outcomes and describing viral suppression patterns among patients receiving standard treatment over a 2-year period.

## Methods

This study was deemed exempt by the University of California, San Francisco Institutional Review Board due to its use of only retrospective clinical data collected during routine care, including a waiver of consent. This report follows the Standards for Quality Improvement Reporting Excellence (SQUIRE) 2.0 guidelines.

### Setting

MXM Health Resource Center is a low-barrier clinic providing transitional primary and urgent care for PEH, most of whom lack reliable mobile phone access. To minimize barriers, the center operates via a 6-day-a-week drop-in care model, offering medical and behavioral health services, laboratory testing, and medication management in a neighborhood with a high concentration of PEH. Care is coordinated with Street Health teams (San Francisco Department of Public Health outreach initiative providing targeted medical care to particularly vulnerable PEH) and satellite clinical services at shelters, syringe programs, and other community-based sites.

During the 2023-2024 fiscal year, MXM Health Resource Center served 4424 adults experiencing homelessness or housing instability, most of whom were covered by Medicaid or other publicly funded access programs (eg, Medicare or Healthy San Francisco). Patients were predominantly cisgender men (66%), with 30% cisgender women, and 3% transgender or nonbinary individuals. Of the overall patient population, 28% of individuals identified as Black, 18% as Hispanic or Latine, 52% as White and 30% as other race or ethnicity.

### Sample

This evaluation included 2 analytic samples: (1) all patients receiving LA-ART at MXM Health Resource Center from November 10, 2021, through October 20, 2025 (programmatic evaluation), and (2) all PWH with 2 or more medical clinician visits at MXM Health Resource Center between January 1, 2023, and December 31, 2024 (descriptive cohort), excluding those receiving primary care elsewhere. Participants in the descriptive cohort were classified as receiving LA-ART or standard HIV treatment (daily oral ART when accepted); fewer than 5% declined oral ART prescription due to adherence challenges or competing priorities.

### Intervention

The MXM Health Resource Center’s LA-ART program, which launched in November 2021, was adapted from protocols developed at the Zuckerberg San Francisco General Hospital Ward 86 clinic to facilitate implementation within a community-based setting for PEH.^10,11,12,13^ Eligibility was streamlined to expand access for individuals unable to maintain oral ART while still requiring regular clinical engagement (Box). Standard treatment consisted of daily oral ART when accepted.

Box. **Key Components of Maria X. Martinez Health Resource Center Long-Acting Antiretroviral Therapy Program**

**Eligibility**

- Prior clinical engagement (eg, monthly visits) for 3 or more months^a^
- No requirement for baseline viral suppression; patients with low CD4 and viremia prioritized^a^
- Willingness to undergo monthly injections and laboratory tests
- Consent for outreach to designated associates if injections are delayed^a^
- Extensive preinitiation counseling on injection site reactions and long pharmacokinetic tail

**Referral and Prescribing**

- Referrals initiated by any health care professional via standardized electronic health record (EHR) note template^a^
- Reviewed and prescribed by designated HIV clinical lead and documented via separate EHR template^a^

**Treatment Protocol**

- Direct-to-inject initiation with cabotegravir (600 mg) and rilpivirine (900 mg) intramuscularly (ie, without oral lead-in)
- Monthly maintenance (cabotegravir, 400 mg, and rilpivirine, 600 mg, intramuscularly every 4 weeks)
- Cabotegravir-rilpivirine dosing every 8 weeks restricted to those with 3 or more months of on-time injections and viral suppression^a^
- Lenacapavir, 927 mg, subcutaneously every 6 months (with 2 oral loading doses) added for patients with baseline cabotegravir or rilpivirine resistance, treatment-emergent cabotegravir-rilpivirine failure, and/or frequent care disruptions^a^

**Program Operations**

- Injections generally administered at drop-in clinic (open 6 days a week); administration at partnering sites and patients’ residences (eg, tents or shelters) possible pending program capacity^a^
- Weekly multidisciplinary huddle^a^ to monitor upcoming and overdue injections via EHR lists
- Proactive reminders to patients or associates (mobile phone, text, email, and/or street outreach^a^ if needed)
- Systemwide be-on-the-lookout alerts distributed to Street Health teams if injections are delayed more than 14 days^a^

**Medication Logistics**

- Single partnering pharmacy delivers medications weekly to clinic
- Medications covered by government insurance; on-site eligibility staff support with insurance gaps
- Medications refrigerated as needed; mobile administration protocol maintains cold chain^a^

**Monitoring and Support**

- Baseline laboratory tests, followed by monthly HIV viral load testing until suppression and quarterly thereafter
- HIV RNA genotype determined at baseline, during viremia, and when any doses are delayed more than 14 days
- $10 gift card incentive for injections and laboratory tests

^a^
May be unique to a status-neutral, homeless health care model compared with HIV specialty clinics.

LA-ART referrals and prescriptions are standardized through electronic health record (EHR) templates (eTables 1-4 in Supplement 1). While any health care professional may refer a patient, to manage program capacity and limit resistance risks all referrals are reviewed by a designated HIV clinical lead physician, who orders medications, after an in-person evaluation when feasible. Selection for LA-ART was based on clinical and programmatic factors, including adherence challenges with oral ART, patient preference, feasibility of injection delivery, and multidisciplinary input (Box).

Treatment follows a direct-to-inject approach, defined as initiation of injections without an oral lead-in, with cabotegravir-rilpivirine administered monthly. Although the US Food and Drug Administration has approved dosing once every 8 weeks, MXM Health Resource Center restricts this option to patients demonstrating reliable adherence and virologic suppression for at least 3 to 6 months; this staged approach supports early retention and engagement. Since April 2023, MXM Health Resource Center has added subcutaneous lenacapavir, dosed every 6 months (with 2 oral loading doses at initiation), as a rescue strategy for patients with virologic failure during treatment with cabotegravir-rilpivirine or baseline cabotegravir or rilpivirine resistance.

Program operations emphasize structured tracking and outreach by a multidisciplinary team, which grew organically from existing staff who expressed interest and capacity. The current team, comprising a lead physician, 2 clinic-based nursing leads, and 2 to 3 outreach staff members (each devoting 10% to 15% full-time equivalents), meets weekly to review upcoming injections via a shared EHR list, coordinate reminders through multiple channels, and mobilize outreach for patients whose injections are overdue. Patients more than 14 days late for injections are flagged with be-on-the-lookout notifications circulated to citywide Street Health teams. Medication logistics are coordinated through a single partnering pharmacy that provides weekly deliveries for on-site refrigerated storage. Outreach staff maintain the cold chain when out-of-clinic administration is necessary. While most individuals are insured through Medicaid, which covers LA-ART in California, coverage gaps are frequent and addressed with on-site eligibility staff. Because many patients experience intermittent incarceration or hospitalization, the program coordinates with local jail health services to continue injections or use temporary oral ART bridging when LA-ART is unavailable (eMethods in Supplement 1).

Laboratory monitoring includes measurement of baseline HIV viral load, CD4 count, and hepatitis B serology, with viral load testing monthly until confirmed viral suppression and quarterly thereafter. HIV RNA genotype is identified prior to baseline and during viremia or late injections. A $10 gift card incentive, introduced in 2023, supports adherence to injections and protocolized laboratory blood draws. The incentive was initially supported by a small grant and subsequently by municipal funds. Together, these strategies—structured referral pathway, direct-to-inject initiation, staged cabotegravir-rilpivirine dosing, rescue with lenacapavir, intensive outreach, and gift card incentive—comprise a multicomponent implementation strategy for delivering LA-ART within a drop-in safety-net clinic.

### Measures

For the LA-ART programmatic evaluation, outcomes assessed feasibility, fidelity, and sustainability, including adoption (number of patients initiating LA-ART), retention (median duration and the proportion of patients continuing to receive LA-ART through October 20, 2025), timeliness (proportion of cabotegravir-rilpivirine injections delivered within the recommended 14-day window), locations at which injections were administered (eg, MXM Health Resource Center or elsewhere), and any necessary protocol adaptations over time. Clinical outcomes for this group included sustained viral suppression, episodes of treatment-emergent cabotegravir or rilpivirine resistance, and cases of lenacapavir rescue.

For the descriptive cohort, sociodemographic and clinical characteristics were included to contextualize the populations. Age, sex, gender identity, race and ethnicity, housing status, psychiatric diagnoses, and substance use disorders were abstracted from the EHR. Race and ethnicity were based on self-report and defined according to standard EHR classifications; race categories were Black, White, and other (which included Asian, American Indian or Alaska Native, Native Hawaiian or Other Pacific Islander, and multiracial), while ethnicity categories were Hispanic or Latine and non-Hispanic or non-Latine. Housing status was defined as street homelessness, sheltered homelessness, or unstably housed, based on documentation from the most recent encounter for patients receiving standard treatment and the time of initiation for patients receiving LA-ART. Clinical outcomes included viral suppression (HIV RNA<200 copies/mL) measured at any time during the 2023-2024 observation period and at the last available measurement within the same period, reflecting routine clinical care with variable testing intervals in this psychosocially complex population. Clinic engagement was measured as the mean number of encounters per person-year.

Data were abstracted from the EHR by 2 clinical team members (L.C. and K.B.O.) using both structured fields (eg, demographics, laboratory values, and problem lists) and clinical documentation. Any discrepancies or unclear documentation were resolved through discussion and review by a third clinical team member (N.J.M.).

### Statistical Analysis

Descriptive statistics (means and SDs for continuous variables; counts and percentages for categorical variables) characterized demographics, clinic engagement, viral suppression, and programmatic outcomes. Program trajectories were examined by reporting cumulative LA-ART outcomes from November 2021 through October 2025, with qualitative descriptions of virologic failure and lenacapavir rescue cases.

To account for treatment transitions between oral ART and LA-ART between 2023 and 2024, viral suppression and clinical encounters were calculated using person-time denominators (person-months aggregated to person-years). Given the nonrandomized design and differences in clinical follow-up, comparisons between treatment groups were descriptive and not intended to estimate causal associations, and formal hypothesis testing was not emphasized to avoid overinterpretation. Analyses were performed using Stata version 18 (StataCorp).

## Results

Between January 2023 and December 2024, 128 PWH received care at MXM Health Resource Center and met inclusion criteria. Of these individuals, 123 received standard treatment and 24 LA-ART; 19 transitioned from oral ART to LA-ART during the observation period. In the LA-ART group, the mean (SD) age was 44.6 (12.2) years and included 15 cisgender males (63%), 3 cisgender females (13%), and 6 transgender or nonbinary individuals (25%). In the standard treatment group, the mean (SD) age was 42.7 (12.2) years and included 69 cisgender males (66%), 16 cisgender females (15%) and 19 transgender or nonbinary individuals (18%) (Table 1). The racial and ethnic composition of the cohort was as follows: 38% (n = 49 of 128) identifying as Black, 30% (n = 38) as White, and 32% (n = 41) as other (ie, Asian, American Indian or Alaska Native, Native Hawaiian or Pacific Islander, or multiracial) and 20% (n = 26) as Hispanic or Latine and 80% (n = 102) as non-Hispanic or non-Latine.

**Table 1.** Demographic and Clinical Characteristics of Patients With HIV Treated Between January 2023 and December 2024^a^

Characteristic	Patients by treatment category, No. (%)	
	Standard treatment only (n = 104)	LA-ART (n = 24)
Age, mean (SD), y	42.7 (12.2)	44.6 (12.1)
Gender		
Cisgender male	69 (66)	15 (63)
Cisgender female	16 (15)	3 (13)
Transgender or nonbinary	19 (18)	6 (25)
Race		
Black	38 (37)	11 (46)
White	33 (32)	5 (21)
Other^b^	33 (32)	8 (33)
Ethnicity		
Hispanic or Latine	19 (18)	7 (29)
Non-Hispanic or non-Latine	85 (82)	17 (71)
Housing status^c^		
Street homelessness	26 (27)	7 (29)
Sheltered homelessness	31 (32)	12 (50)
Unstably housed (eg, SRO or PSH)	41 (42)	5 (21)
Any mental health diagnosis	64 (62)	17 (71)
Primary psychotic disorder	28 (27)	9 (38)
Any substance use disorder	81 (78)	20 (83)
Stimulant use disorder	68 (66)	20 (83)
Opioid use disorder	29 (28)	6 (25)
Viral suppression at time of LA-ART initiation	NA	6 (25)

Abbreviations: LA-ART, long-acting antiretroviral therapy; NA, not applicable; PSH, permanent supportive housing; SRO, single-room occupancy.

^a^ Columns are dichotomized based on any receipt of LA-ART during the observation period.

^b^ Other race includes those identifying as Asian, American Indian or Alaska Native, Native Hawaiian or Pacific Islander, or multiracial.

^c^ Housing status reflects the last encounter for patients receiving standard treatment and the time of initiation of injections for patients receiving LA-ART.

Other baseline characteristics of patients receiving exclusively standard HIV treatment vs any LA-ART over the 2-year period are listed in Table 1. All patients were experiencing homelessness or housing instability, with a high prevalence of substance use disorders and primary psychotic disorders.

Mean (SD) clinical encounters per person-year were 23.0 (11.1) among those receiving LA-ART and 9.1 (11.5) among those receiving standard treatment (Table 2). Seventy-five percent (n = 18 of 24) of patients receiving LA-ART had HIV viremia at baseline, and all 24 (100%) achieved and/or maintained viral suppression during the observation period. Among patients receiving standard treatment, 89 of 123 (72%) achieved viral suppression at least once during the observation period, and 69 of 123 (56%) had viral suppression at last follow-up.

**Table 2.** Clinical Encounters and Viral Suppression by Treatment Between January 2023 and December 2024

Outcome	Standard treatment	LA-ART
Total visits during study period	1462	750
Total person-years	161.5	32.6
Clinic visits per person-year, mean (SD)	9.1 (11.5)	23.0 (11.1)
Patients with viral suppression at any time, No. (%)^a^	89 of 123 (72)	24 of 24 (100)
Patients with viral suppression at last follow-up, No. (%)^a^	69 of 123 (56)	24 of 24 (100)

Abbreviation: LA-ART, long-acting antiretroviral therapy.

^a^ Viral suppression was defined as HIV RNA less than 200 copies/mL.

### LA-ART Program Evaluation

Since program launch in November 2021 through October 2025, 30 PWH initiated LA-ART, with follow-up ranging from 8 to 206 weeks (median, 106 weeks). Of these individuals, 22 (73%) had viremia at LA-ART initiation, including 15 (50%) who lacked documented viral suppression for the 3 or more years prior. All continued to receive LA-ART through October 20, 2025, with 6 transitioning to new HIV primary care homes. In addition, 10 LA-ART recipients also received 1 or more other long-acting injectable medications, including antipsychotics, buprenorphine, and hormone therapies, with most having started LA-ART prior to receiving the other injectables.

A total of 637 injection visits were completed, of which 43 (7%) were delayed by more than 7 days, including 21 (3%) delayed more than 14 days, which required medication reloading. While most injections (81%) were administered at MXM Health Resource Center, 121 (19%) were delivered within shelters, tents, syringe access programs, hospitals, or jails (Table 3). Ninety-one injections administered in shelters or tents were delivered to 7 patients, 5 of whom routinely received their doses outside the clinic due to psychological challenges engaging within clinic settings.

**Table 3.** Programmatic Outcomes for Patients Receiving LA-ART From November 2021 Through October 2025

Outcome	No. (%)
Patients receiving LA-ART	
No.	30
Follow-up duration, median (range), wk	106 (8-206)
Retention as of October 20, 2025^a^	30 (100)
Transitioned to new HIV primary care	6 (20)
Injection visits completed	
No.	637 (100)
Delayed >7 d	43 (7)
Delayed >14 d	21 (3)
Administered at Maria X. Martinez Health Resource Center	514 (81)
Administered in shelter or tent	91 (14)
Administered in jail or hospital	11 (2)
Administered at syringe access site	21 (3)
Virologic failure with emergent resistance during cabotegravir-rilpivirine^b^	
No.	3
Resuppression with addition of LEN	3 (100)
Current LA-ART regimen	
Cabotegravir-rilpivirine every mo	13 (43)
Cabotegravir-rilpivirine every 2 mo	8 (27)
Cabotegravir-rilpivirine every mo plus lenacapavir every 6 mo	7 (23)
Cabotegravir every mo plus lenacapavir every 6 mo	2 (7)

Abbreviation: LA-ART, long-acting antiretroviral therapy.

^a^ Retention defined as 1 or more clinical visits at the Maria X. Martinez Health Resource Center or a new HIV primary care site within 2 months prior to database closure (October 2025).

^b^ Virologic failure defined as HIV RNA 200 copies/mL or greater on 2 or more consecutive measurements after prior suppression.

### Program Adaptations and Outcomes

Over time, the LA-ART program modified its protocol to improve operational efficiency. Modifications included (1) a requirement for patients to have engaged with MXM Health Resource Center clinical staff for 3 or more encounters over the 3 months preceding LA-ART initiation to ensure trust and rapport, (2) implementation of a $10 gift card incentive for injections and adherence to protocolized laboratory blood draws, and (3) introduction of lenacapavir as a rescue agent for patients with virologic failure and those with evidence of cabotegravir or rilpivirine resistance on baseline genotype, as well as those anticipated to have care disruptions due to severe mental illness. These adaptations were informed in part by the experiences of 3 of 30 patients receiving LA-ART (10%) who developed treatment-emergent resistance within the first 6 months of initiating cabotegravir-rilpivirine (Table 4). None of these patients had achieved viral suppression with oral ART for 3 or more years prior to initiating injections, and all had substantial psychiatric and substance use comorbidities. Each patient declined oral ART after virologic failure during cabotegravir-rilpivirine treatment but achieved resuppression and CD4 recovery with lenacapavir-based regimens. A fourth patient experienced a single episode of viremia (approximately 700 copies/mL) during cabotegravir-rilpivirine treatment, without confirmed resistance due to unsuccessful RNA genotyping; lenacapavir was added, and suppression has been maintained for more than 114 weeks.

**Table 4.** Characteristics and Outcomes of Patients Requiring Lenacapavir Rescue After Cabotegravir-Rilpivirine Failure From November 2021 Through October 2025

Patient^a^	Factors associated with cabotegravir-rilpivirine failure^b^	Emergent resistance^c^	Outcome after cabotegravir-rilpivirine failure^d^	CD4 count, cells/μL (baseline and latest)^e^	Notes
A	Delayed injections due to loss of temporary housing (12 and 21 d overdue)	Rilpivirine: K101P, K103N, V108I, E138K, and Y181C; cabotegravir: E138K, G140S, and Q148R	Resuppression with cabotegravir-rilpivirine plus lenacapavir for >105 wk	187 and 554	3 subsequent delayed cabotegravir-rilpivirine doses (13, 21, and 17 d overdue)
B	Transiently declined injections due to mental health challenges (83 d overdue)	Rilpivirine: V179D, M230, and Y181C	Resuppression with cabotegravir plus lenacapavir for >131 wk	18 and 412	1 subsequent delayed cabotegravir dose (14 d overdue)
C	Suspected subcutaneous (ie, not intramuscular) administration of initial injection due to limited access to injection site during mobile outreach (no overdue doses)	Rilpivirine: V106I and Y181C	Suppression with cabotegravir plus lenacapavir for >74 wk	18 and 227	All injections administered at patient’s residence

^a^ Three patients developed treatment-emergent resistance to cabotegravir-rilpivirine and had successful rescue with lenacapavir-based regimens.

^b^ Virologic failure defined as HIV RNA 200 copies/mL or greater on 2 or more consecutive measurements after prior suppression.

^c^ Resistance sequence variants identified on HIV-1 RNA genotypes.

^d^ Follow-up reflects duration of subsequent viral suppression with lenacapavir-containing regimens through October 2025.

^e^ CD4 counts reported as baseline (prior to long-acting antiretroviral initiation) to the latest available.

## Discussion

This quality improvement study extends prior work from specialty HIV clinics by demonstrating the sustained feasibility and successful outcomes of LA-ART for PWH experiencing homelessness or housing instability within a municipal drop-in clinic serving people who have difficulty engaging in traditional appointment-based care. Over 4 years, the program achieved high retention and durable viral suppression with LA-ART despite high rates of psychiatric illness, substance use disorders, and structural vulnerability—factors that have historically excluded patients from clinical trials and limited the reach of HIV specialty care.^17,18,19,20^

Viral suppression was high among patients receiving LA-ART, with 100% maintaining suppression at the latest available follow-up. Only 56% of patients receiving standard treatment alone had viral suppression at the end of the 2-year observation period. Importantly, these groups are not directly comparable, as findings reflect both pharmacologic differences in treatment approach as well as program structure. Adherence to eligibility criteria, standardized referral processes, weekly team huddles, and multimodal outreach were critical to the LA-ART program’s operations, with nearly 1 in 5 injections administered outside of the clinic and requiring mobile nursing support. This finding suggests that successful LA-ART delivery for highly marginalized populations depends not only on medication availability but also on flexible, continuous, and harm reduction–oriented systems of care.^10,11,12,13^

While mobile delivery may be challenging to replicate in many safety-net settings, it was needed routinely for only a small subset of patients who were otherwise unable to engage in clinic-based care. Therefore, low-barrier clinics lacking mobile capacity may still be able to offer LA-ART, although some patients may remain difficult to engage without this flexibility.

The greater frequency of clinical encounters necessary for LA-ART recipients, driven by required injection visits every 1 to 2 months compared with the expectation of quarterly visits for those receiving oral ART, may pose challenges for patients with competing priorities. However, these encounters also increase opportunities to address other health care needs, including screening and treatment for sexually transmitted infections, substance use disorders, and psychiatric conditions. One-third of patients receiving LA-ART at MXM Health Resource Center also received other long-acting injectable therapies, suggesting that this program may provide effective scaffolding for treating comorbid conditions.

Our experience also highlights important risks and program adaptations over time. Three patients with longstanding viremia and co-occurring substance use and psychiatric disorders developed treatment-emergent cabotegravir or rilpivirine resistance. However, they subsequently achieved resuppression with CD4 recovery after lenacapavir-based rescue therapy. These and other cases of patients initiating cabotegravir-rilpivirine plus lenacapavir at baseline (due to historical resistance) have informed additional protocol adaptations, including use of cabotegravir-rilpivirine plus lenacapavir for select patients at the highest risk of care interruptions. Prior findings suggest that some patients may maintain viral suppression despite unintended periods of lenacapavir monotherapy,^21^ although this approach requires further study.

### Limitations

Study limitations include the single-site design, modest sample size, and nonrandomized, descriptive analysis. Patients initiating LA-ART were selected based on program criteria, including preexisting engagement with clinic staff and completion of preinitiation counseling—factors that may limit generalizability to less-engaged populations and indicate that individuals receiving LA-ART may differ systematically from those receiving standard treatment. Program costs, including staff time, outreach, pharmacy coordination, and sustainability of incentive funding, were not systematically captured. Results also reflect a setting with robust Medicaid coverage of LA-ART, and changes in eligibility policies may affect continuity of access.^22,23^ Even short interruptions in LA-ART may increase risk of resistance due to the long pharmacokinetic tails of cabotegravir, rilpivirine, and lenacapavir, underscoring the importance of program infrastructure to support continuity. Accordingly, these findings should be interpreted as proof of concept for a high-touch delivery model rather than evidence supporting universal use of LA-ART in this population.

## Conclusions

This quality improvement study complements larger analyses from HIV specialty clinics by demonstrating that, when paired with intensive, flexible support, LA-ART can be delivered in a nonspecialty, community-based setting serving PEH. These findings highlight the potential role of low-barrier clinics in extending access to LA-ART for populations with substantial barriers to oral ART and appointment-based care and in advancing public health goals, such as the global Getting to Zero initiative aiming for zero new HIV infections, by engaging the hardest-to-reach individuals whose virus remains unsuppressed. However, implementation requires significant programmatic support, and findings should be interpreted in the context of a resource-intensive model. Future work should examine cost, scalability, and implementation strategies to identify which program components are most essential for successful adaptation across safety-net settings.

## Notes

Supplement 1. **eTable 1.** LA-PrEP Dosing Schedules **eTable 2.** LA-PrEP Traffic Light Tables **eTable 3.** LA-ART Dosing Schedules **eTable 4.** LA-ART Traffic Light Tables **eMethods.** General LAI Logistics, LA-PrEP Protocols, LA-ART Protocols, SmartPhrases

Supplement 2.
Data Sharing Statement
