# Supplementary material for: Long-Acting Antiretroviral Therapy for HIV via Drop-in Community-Based Care
Source: JAMA Netw Open. 2026 Jun 26;9(6):e2620348. doi: 10.1001/jamanetworkopen.2026.20348 (PMC13309861; doi:10.1001/jamanetworkopen.2026.20348)
Supplement: Supplement 1. — eTable 1. LA-PrEP Dosing Schedules eTable 2. LA-PrEP Traffic Light Tables eTable 3. LA-ART Dosing Schedules eTable 4. LA-ART Traffic Light Tables eMethods. General LAI Logistics, LA-PrEP Protocols, LA-ART Protocols, SmartPhrases [file jamanetwopen-e2620348-s001.pdf]

Supplemental Online Content

Mehtani NJ, Matzat SJ, O'Connor K, et al. Long-acting antiretroviral therapy for HIV via drop-in community-based care. *JAMA Netw Open*. 2026;9(6):e2620348.
doi:10.1001/jamanetworkopen.2026.20348

eTable 1. LA-PrEP Dosing Schedules

eTable 2. LA-PrEP Traffic Light Tables

eTable 3. LA-ART Dosing Schedules

eTable 4. LA-ART Traffic Light Tables

eMethods. General LAI Logistics, LA-PrEP Protocols, LA-ART Protocols, SmartPhrases

This supplemental material has been provided by the authors to give readers additional information about their work.

© 2025 Mehtani NJ et al. *JAMA Network Open*.

LA-PrEP Quick Reference

eTable 1. LA-PrEP Dosing Schedules

	Cabotegravir (Apretude)	Lenacapavir (Yeztugo)
**Loading**	600mg IM x2 (4 weeks apart)	Day 1: 927mg SQ + 600mg PO<br>Day 2: 600mg PO (out of clinic)
**Maintenance**	600mg IM q8 weeks (±7 days)	927mg SQ q26 weeks (±2 weeks)
**Injection Site**	Gluteal (ventrogluteal preferred)	Abdomen (thigh as alternative)
**Labs* (during maintenance phase)**	HIV Ag/Ab (**recommended**) + HIV POCT (**required**)	HIV Ag/Ab **(required)** + HIV POCT **(required)**

***WPIC Practice Note:** Due to high HIV risk in our population, HIV RNA VL (rather than an HIV Ag/Ab test) is recommended at baseline (to confirm HIV negative status prior to initiating LA-PrEP) and whenever a patient is >3 months overdue for a maintenance dose.*

eTable 2. LA-PrEP Traffic Light Tables

Cabotegravir (Apretude) for PrEP	
**Days since last injection**	**Action**
**≤ 63 Days** (≤35 if loading dose)<br>(up to 7 days overdue)	1. Administer CAB 600mg IM. *Next dose in 8 weeks.*<br>2. Draw HIV Ag/Ab (recommended) + POCT (required).
**64 - 84 Days** (36-56 if loading dose)<br>(8 -28 days overdue)	1. Administer CAB 600mg IM. *Next dose in 8 weeks.*<br>2. **MANDATORY:** Draw HIV Ag/Ab + POCT (must be negative).
**>84 Days** (>56 if loading dose)<br>(>28 days overdue)	**STOP.**<br>1. Obtain HIV Ag/Ab + POCT.<br>2. Restart **Loading Sequence** (2 doses, 4 weeks apart).

Lenacapavir (Yeztugo) for PrEP	
**Days since last injection**	**Action**
**24 - 28 Weeks**<br>(up to 2 weeks overdue)	1. Administer LEN 927mg SQ. *Next dose in 26 weeks.*<br>2. **MANDATORY:** Draw HIV Ag/Ab + POCT (must be negative).
**>28 Weeks**<br>(>2 weeks late)	**STOP.**<br>1. Obtain HIV Ag/Ab + POCT.<br>2. Restart **Loading Sequence** (SQ+PO Day 1, PO Day 2). *Next dose in 26 weeks.*

LA-ART Quick Reference

eTable 3. LA-ART Dosing Schedules

	CAB/RPV q4-wk	CAB/RPV q8-wk	LEN (Sunleca)
**Loading**	600/900mg IM × 1	600/900mg IM q4 wks × 2	Day 1: 927mg SQ + 600mg PO<br>Day 2: 600mg PO
**Maintenance**	400/600mg IM q4 week	600/900mg IM q8 week	927mg SQ q26 week
**Eligibility**	Default regimen	After ≥3 months on-time q4-week dosing	Added to CAB/RPV if there is baseline CAB or RPV resistance, or if patient is at high risk of care interruptions

eTable 4. LA-ART Traffic Light Tables 🚦

**CAB 400 + RPV 600 (Cabenuva) for ART - q4-week**	
**Days since last injection**	**Action**
**≤ 37 Days**<br>*Up to 9 days overdue*	1. Administer Maintenance Dose (400/600mg). *Next dose in 4 weeks.*<br>2. **RECOMMEND**: HIV VL q3-6 months
**38 - 42 Days**<br>*10 - 14 days overdue*	1. Administer Maintenance Dose (400/600mg). *Next dose in 4 weeks.*<br>2. **MANDATORY**: Draw HIV VL
**> 42 Days***<br>*15+ days overdue*	🛑 **STOP. Consult Provider.**<br>1. **MANDATORY**: Draw HIV VL + Genotype<br>2. Restart **Loading Dose** (600/900mg x1), then return to q4-wk 400/600mg

**CAB 600 + RPV 900 (Cabenuva) for ART - q8-week**	
**Days since last injection**	**Action**
**≤ 65 Days**<br>*up to 9 days overdue*	1. Administer Maintenance Dose (600/900mg). *Next dose in 8 weeks.*<br>2. **RECOMMEND**: Draw HIV VL q3-6 months
**66 - 70 Days**<br>*10 - 14 days overdue*	1. Administer Maintenance Dose (600/900mg). *Next dose in 8 weeks.*<br>2. **MANDATORY**: Draw HIV VL
**> 70 Days***<br>*15+ days overdue*	🛑 **STOP. Consult Provider**<br>1. **MANDATORY**: Draw HIV VL + Genotype.<br>2. Restart **Loading Sequence** (600/900mg q4wk x2), then return to q8-wk

**LEN (Sunleca) for ART**	
**Days since last injection**	**Action**
**24 - 28 Weeks**<br>*up to 2 weeks overdue*	1. Administer LEN 927mg SQ.<br>2. **RECOMMEND**: Draw HIV VL q3-6 months
**>28 Weeks**<br>*>2 weeks overdue*	🛑 **STOP. Consult Provider**. Check for adherence to other ART agents.<br>1. **MANDATORY:** Draw HIV VL + Genotype.<br>2. Restart **Loading Sequence** (SQ+PO Day 1, PO Day 2), then return to q26-wk

** **WPIC Practice Note:** These thresholds are more conservative than FDA labeling, which permits maintenance dosing continuation for up to 8 weeks or 12 weeks since the last injection for patients on CAB/RPV q4-week or q8-week dosing regimens, respectively, before requiring a reload. A lower threshold is applied at MXM given that most of our patients are viremic at baseline and at higher risk for repeated dosing delays.*

eMethods.

1. General LAI Logistics

This section covers operational workflows applicable to all LAI antiretroviral regimens.

1.1 Storage & Handling

- **CAB/RPV (Cabenuva) - refrigerated**
    - Store in medication refrigerator until patient arrives.
    - Remove from refrigerator and allow to reach room temperature for **minimum 15 minutes** before injection.
    - Maximum time out of refrigeration: **6 hours.**
    - *For outreach*: Transport in resealable plastic bag with ice packs; label with "Time Removed." Return to refrigerator if patient not located.
- **CAB (Apretude) & Lenacapavir - room temperature**
    - Store at 20–25°C; excursions permitted to 15–30 °C.
    - No refrigeration required.

1.2 Injection Site Reaction (ISR) Mitigation

Note: ISRs are common (~75-80% report mild pain) but often improve with subsequent injections.

- **Analgesia options:**
    - Ice: 10 mins pre-injection.
    - Lidocaine: Topical cream 30 mins pre-injection.
    - Analgesia: APAP/NSAIDs.
- **Technique:**
    - Pause 5 seconds before withdrawing needle to prevent leakage.
    - Needle size (CAB and CAB/RPV):
    - 1.5-inch needle for patients <90kg and BMI < 30.
    - 2-inch needle for patients ≥ 90kg or BMI ≥ 30.
    - Follow injection instructions provided by manufacturer, including Z-track method for intramuscular injections (CAB and CAB/RPV)

1.3 Tracking & Outreach Workflows

- **Weekly ETE Huddle**
    - Attendees: AmeriCorps fellow, QI RN Lead, MXM Clinic RN, Street Med RN, ETE HW III, ETE LVN, HIV Clinical Lead MD
    - Review: all patients with injections due within 2 weeks or recently missed
- **Outreach Protocol**
    - **Patients due within 1–12 days:**
        - Phone call to patient and/or close contacts
        - Coordinate mobile outreach as needed
    - **Patients overdue:**
        - Mobile street-based outreach
        - Phone calls to all emergency contacts
        - Engagement with case managers and partnering CBOs
        - Review ONE system for shelter moves
        - Review VineLink.com for out-of-county incarceration

        - If >14 days late: BOLO ("Be On Look Out") with patient photo sent to WPIC Street Medicine/Shelter Health listservs

- **Outreach Intensity**
    - LA-ART patients: Outreach continues until patient is located.
    - LA-PrEP patients: 3 outreach attempts (phone, in-person, or through contacts). Medication held at MXM for 3 months before discarding.
- **Medication Delivery**
    - Following weekly huddle, MXM Clinic RN sends medication list to AHF Pharmacy.
    - Medications delivered to MXM on Thursdays.
    - LA-ARVs ordered up to 1 week in advance of due date to allow early injection if desired.
- **Epic Documentation**
    - Shared Patient List: Maintained by ETE team; tracks all LA-ART/LA-PrEP patients
    - Blue Sticky Notes: Next due date and lab requirements
    - MAR: Both AMB (ambulatory) and CAM (clinic-administered medication) orders required

1.4 Incentives

To promote adherence, staff may dispense gift cards on injection visits (as available):

- Lab draw: $10
- CAB or CAB/RPV injections: $10
- Lenacapavir injection: $20

1.5 Special Circumstances

In the event that a patient is hospitalized or incarcerated at the time that an injection is due, notify clinical staff at the site to:

- Restart previous suppressive daily oral ART (or DRV/c/TAF/FTC if unknown) or oral PrEP (TDF/FTC) immediately.
- Request permission for ETE team to bring LA-ART/LA-PrEP to hospital for administration.
- For SF County Jail: Coordinate medication courier from MXM through Jail Health Services.
- If injection cannot be given during hospitalization/incarceration, continue oral regimen and plan to administer the injection on the day of or after discharge.

2. LA-PrEP Protocols

Any provider may initiate patients on long-acting PrEP. The **.LAPREP** SmartPhrase explains how to order IM Cabotegravir (Apretude) or SQ Lenacapavir (Yeztugo) as LA-PrEP. Please note that while MediCal covers both LA-PrEP options, several private insurers and Medicare Part D *do not*, so please verify insurance coverage prior to ordering.

2.1 Eligibility Checklist

*See **.LAPREP** SmartPhrase.*

2.2 Lenacapavir Drug-Drug Interactions

Drug Class	Specific Agents	Action
Anticonvulsants	Carbamazepine, Oxcarbazepine, Phenytoin, Phenobarbital	🛑 STOP. Absolute Contraindication.<br>Do not use LEN.<br>Levels will be subtherapeutic and can lead to resistance.
Antimycobacterials	Rifampin, Rifabutin, Rifapentine	🛑 STOP. Absolute Contraindication.<br>Do not use LEN.
Opioids/Benzos/ Ketamine/Alcohol	Fentanyl, Methadone, Alprazolam (Xanax), ketamine	⚠ PROCEED WITH CAUTION.<br>LEN increases these drug levels.<br>Counsel patient: *"This shot makes your drug supply stronger. Use less than usual–especially over the next few days–to reduce overdose risk."*
Erectile Meds	Sildenafil (Viagra), tadalafil (Cialis)	⚠ PROCEED WITH CAUTION.<br>LEN increases these drug levels.<br>Instruct patient to reduce ED medication dose by 50%.

2.3 Counseling Points

- **General PrEP Counseling:** LA-PrEP should be offered within the broader menu of HIV prevention strategies, including discussion of relative benefits, limitations, and follow-up requirements for each option.
    - See here for patient-facing material to aide discussions of options.

TDF/FTC	TAF/FTCⓘ	2-1-1 TDF/FTCⓘ	Cabotegravir	Lenacapavir
Truvada	Descovy	Truvada	CAB-IM, Apretude	LEN-SQ, Yeztugo

ⓘif cis man and trans woman who has sex with men
**eGFR must be >60 mL/min for TDF- or >30 mL/min for TAF-containing regimen*

- **LA-PrEP Options: CAB-IM (Apretude) and LEN-SQ (Yeztugo)**
    - Adherence to injection schedule is required to minimize HIV risk and resistance.
    - Regular HIV/STI testing is required before initiation and with each injection visit.
    - Contingency planning if doses are delayed or LA-PrEP is discontinued.

- Injection site reactions are common (pain, redness, swelling, firmness, nodules); usually mild-moderate and improve with subsequent doses.

- **The "Tail"**
    - *“After stopping injections, medication remains in your body for up to 12 months. After [8 weeks for CAB / 6 months for LEN], drug levels drop too low to protect you from HIV but remain high enough to cause drug resistance if you do catch HIV. If you miss a shot or stop treatment, you must use condoms or switch to oral PrEP to prevent HIV infection.”*

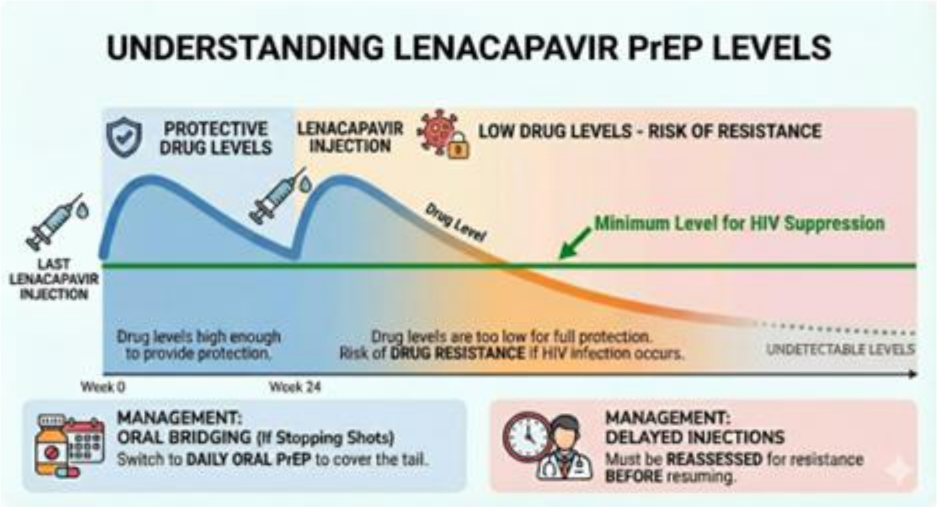

- **Lenacapavir-Specific DDI-Counseling: Opioids, Benzodiazepines, PDE-5 Inhibitors**
    - *"This drug can make opioids (like fentanyl), benzodiazepines (like Xanax), and erectile dysfunction medications (like Viagra or Cialis) much stronger and last longer. Please use less than your usual amount—or half the dose of any ED medications—after receiving the shot to see how it affects you. We don't want you to overdose."*

- **Pregnancy and Lactation**
    - Based on currently available limited human data, both LA-PrEP options are not thought to pose risks for fetal development or lactation. However, more data are available for LEN (Yeztugo) during pregnancy and lactation than for CAB (Apretude).
    - Pregnancy Exposure Registry: Encourage registration with the Antiretroviral Pregnancy Registry at 1-800-258-4263 if pregnancy occurs.
    - Breastfeeding: Lenacapavir is present in human milk at very low levels; adverse effects in breastfed infants have not been observed.
    - Recommend offering discussion around reproductive health for patients capable of pregnancy.

2.4 Lab Monitoring for LA-PrEP

**Baseline** (within 7 days first injection)	**Maintenance**	**Post-Discontinuation**
**<u>Required</u>:**<br>• HIV VL*<br>• POC HIV Ag/Ab (if same-day start)**<br>• Syphilis serology<br>• 3-site GC/CT NAAT<br>• Trich (if vagina present)<br>• M-gen (if symptomatic)<br>• Urine pregnancy (if pregnancy-capable)<br><br>**<u>If none in last 12 months</u>:**<br>• Hep B serologies (HBsAg / HBsAb / HBcAb)<br>• HCV (antibody vs VL)<br>• CMP	**<u>Every injection visit</u>:**<br>• HIV Ag/Ab (blood draw rec'd)<br>• POCT HIV Ag/Ab is an acceptable alternative ONLY if both:<br>- On-time (or <4 wks late on CAB)<br>- Ag/Ab drawn within last 6 months<br><br>**<u>If LATE for injection:</u>**<br>• CAB >4 wk late: MUST draw Ag/Ab + POCT<br>• LEN >2 wk late: MUST draw Ag/Ab + POCT<br><br>**<u>q2-4 mo. (if sexually active)</u>:**<br>• Syphilis serology<br>• 3-site GC/CT NAAT<br>• Trich (if vagina); M-gen (if symptomatic)<br>• Urine pregnancy	• HIV Ag/Ab q3 months × 12 months<br>• comprehensive STI screening at d/c time and as clinically indicated

***HIV RNA VL at baseline:** While HIV Ag/Ab (without viral load) is permissible at baseline based on some clinical guidelines, WPIC recommends HIV VL at LA-PrEP initiation and with late maintenance doses (e.g., >3 months late) given the high prevalence of HIV sexual risk factors in our population.*

***** POCT HIV:** An acceptable same-day start option for initiating either CAB or LEN, but this must be paired with HIV RNA VL if the patient has any recent possible HIV exposures (within the past 21 days).*

2.5 Ordering Workflow

All new LA-PrEP orders require three components:

- AMB Order (e-Rx): Send to AHF Pharmacy with .AHFNOTE SmartPhrase in "Notes to Pharmacy."
- CAM Order: Select "Patient supplied medication."
- Referral Note: Route note with **.LAPREP** to DPH AMB WPIC ETE POOL.

Use SmartSet: DPH AMB HIV LAI (includes all required orders).

2.6 Discontinuing LA-PrEP

Both CAB and LEN have a prolonged and variable PK "tail." If considering discontinuing LA-PrEP:

- Offer comprehensive STI screening at time of discontinuation.
- HIV Ag/Ab testing every 3 months for 12 months after last injection.
- If HIV risk continues, strongly recommend alternative PrEP:
    - Start oral PrEP (TDF/FTC or TAF/FTC) 8 weeks after last CAB injection.
    - Start oral PrEP or CAB-LA 24–26 weeks after last LEN injection.

3. LA-ART Protocols

In contrast to LA-PrEP, initiation of injectable ART is **restricted**. While any provider may **refer** a patient for consideration, only HIV Clinical Leads may initiate treatment after review with the ETE team.

3.1 Referral Process

- Any WPIC MD/NP/RN completes **.LAARTREFERRAL** SmartPhrase.
- Route note to DPH AMB WPIC ETE POOL.
- If eligible, patient should present for in-person visit with HIV Clinical Lead (Mondays at MXM).
- HIV Clinical Lead completes **.LAARTSTART** SmartPhrase to confirm eligibility and order medications

3.2 Eligibility Checklist

- [ ] **Engagement:** Engaged with WPIC >3 months (with at least monthly)
- [ ] **Plan:** Staying in SF for next 12 months.
- [ ] **Clinical Indication:** CD4 <200 and/or persistent viremia due to adherence barriers for ≥1 year
- [ ] **Commitment:** Agrees to monthly injections + lab draws until HIV VL <30 x2 consecutive months, then q3 months
- [ ] No contraindicated resistance mutations (see below).

WPIC Practice Note: *Patients with baseline CD4 >200 who can adhere to oral ART should generally be referred to an HIV Specialty Clinic (e.g., Ward 86, SFCHC) for comprehensive HIV primary care and LA-ART initiation. WPIC resources are limited, with the goal of preventing AIDS-associated morbidity in PEH with no other options.*

3.3 Resistance Review

Review all prior HIV RNA genotypes before initiation. Patients with the following mutations are NOT eligible for CAB/RPV alone (but may be considered for CAB/RPV + LEN on a case-by-case basis):

Drug	Mutations
**RPV**	L100I, K101E/P/Q, E138A/G/K/Q/R, Y181C/I, Y188L, H221Y, P225H, F227C, M230L
**CAB**	T97A, G118R, Q148H/K/R/S, E138A/K, G140A/R/S, N155H, R263K

3.4 Counseling Points

- *"Starting LA-ART is not a casual decision. If you miss injections, the virus can develop resistance to the medication, and you may lose the ability to use these long-acting drugs—and many related oral HIV medications—forever. We need a backup contact person we can call if you miss a planned injection."*
- **Key Points to Cover**
  - LA-ART at WPIC should be considered a permanent/long-term plan.
  - If late for injection: high risk for resistance to two major ARV classes (INSTI and NNRTI), including oral medications.
  - If discontinuing CAB/RPV after initiation: must adhere to daily oral ART for ≥12 months to minimize resistance risks from PK tail.
  - Detectable baseline viremia is acceptable but not ideal; viral suppression on oral ART prior to initiation is encouraged.
  - ISRs are common but generally improve after first dose (see Section 1.2).

- **Contingency Agreement:** if >7 days late for injection, patient must agree to:
    - Restart daily oral suppressive ART (e.g., DRV/c/TAF/FTC) until next injection.
    - Allow ETE team to contact emergency contacts and conduct mobile outreach.

3.5 Lab Monitoring for LA-ART

**Baseline**<br>(within 7 days of first injection)	**Until Suppressed**<br>(VL <30 × 2)	**Maintenance**
<u>**Required**</u>:<br>• HIV VL<br>• CD4 count<br>• CMP, CBC<br>• HIV Genotype (if none in past 3 months)	<u>**With every injection**</u>:<br>• HIV VL<br><br><u>**With 1st maintenance dose or if VL ≥200 copies**</u>:<br>• HIV Genotype	<u>**Every 3 months**</u>:<br>• HIV VL<br>• STI screens (if ongoing risk)<br><br><u>**Every 3-12 months**</u>:<br>• CD4 (more frequent if <200)<br>• CBC, CMP (as indicated)

Note: We consider HIV RNA <200 copies/mL as suppressive on LA-ART. VL blips <200 copies are common and do not appear to have significant consequences for resistance development.

3.6 Ordering Workflow

HIV Clinical Lead completes the following:

- AMB Orders (e-Rx to AHF Pharmacy):
    - Loading dose: CAB/RPV 600mg/900mg × 1
    - Maintenance doses: CAB/RPV 400mg/600mg × 3 (auto-refills)
    - Include <u>.AHFNOTE</u> in "Pharmacy Instructions"
- CAM Orders:
    - Loading dose: CAB/RPV 600mg/900mg PRN "patient presents for 1st dose"
    - Maintenance doses: CAB/RPV 400mg/600mg q28 days
- Lab Orders:
    - HIV VL × 10 (nurse release)
    - CD4 × 3 (nurse release)
    - HIV RNA Genotype × 1 (if viremic at baseline)
    - STI labs if sexually active (RPR × 10; GC/CT × 10 at appropriate sites)
- Route chart to DPH AMB WPIC ETE POOL.

3.7 Discontinuing LA-ART

- While LA-ART discontinuation is expected to be rare, if a patient decides to stop:
    - Initiate suppressive oral ART regimen within 1 month of last CAB/RPV injection for q4-week patients; or within 2 months for q8-week patients.
    - Continue oral ART for at least 12 months to address resistance risks from PK tail.
    - Consider referral to Health at Home / Treatment Adherence Program for daily observed therapy if needed.

4. SmartPhrases

.LAPREP

WPIC Injectable PrEP Initiation - any provider may use to start injectable PrEP

WPIC INJECTABLE PrEP INITIATION

{Any provider may initiate long-acting PrEP. Please note that while MediCal covers both LA-PrEP options, several private insurers and Medicare Part D do not, so please verify insurance coverage prior to ordering.:30373}
{Link to full protocol:30373}
{Considerations -
CAB- more frequent dosing, IM formulation, no opioid/BZD interaction concerns.
LEN- q6-month dosing, SQ formulation, may increase opioid/BZD levels - use caution:30373}

ELIGIBILITY VERIFICATION

☐ HIV negative: Last Ab/Ag **@LPPLINK(410842,3)@** on **@LPPLINK(410843,3)@**. (Should be within 7 days of first injection, or scheck HIV VL if high-risk exposure in prior 21 days).
☐ Weight: ≥35kg. Last weight **@LPPLINK(22011,3)@**.
☐ Review Drug-Drug Interactions:
- 🛑 Contraindicated: Anticonvulsants (carbamazepine, phenytoin, phenobarbital) or Antimycobacterials (rifampin, rifabutin).
- ⚠ Opioids/Benzos : Caution in first few weeks, especially first few days. (If LEN)
- ⚠ Erectile dysfunction Rx's: Reduce dose of PDE5-inhibitors by 50%. (If LEN)

COUNSELING & CONSENT

☐ Consents to treatment and understands:
- Resistance Risk: Missing injections while sexually active risks acquiring resistant form of HIV.
- Schedule: Plan for visits every 2 months (CAB) or 6 months (LEN).
- Discontinuation: The "Tail" - medication persist for 6-12 months after stopping.

CONTACT INFORMATION

- Location generally sleeps: {housing:19197::"@STREET@","unsheltered - ***","***"}
- Phone/email: {contact:19197::"@PHONE@","@EMAIL@","none","***"}
- Best way to contact for injection reminders: ***
- Community Contacts {friends, partners, case managers, etc:30373}

Name	Relationship	Phone/Email	OK for us to contact if late for injection?
***	***	***	☐ Yes ☐ No
***	***	***	☐ Yes ☐ No {'Ctrl +]' to add row:30373}

PLAN & ORDERING {use DPH AMB HIV LAI SmartSet for orders:30373}

☐ Rx: {medication:19197::"Apretude (Cabotegravir IM: Loading q1m x2 -> Maintenance q2m","Yeztugo (Lenacapavir SQ): Loading Day 1 SQ+PO, Day 2 PO -> Maintenance q6m"}
- eRx to AHF Pharmacy, with .AHFNOTE in 'Notes to Pharmacy'
- CAM order, with 'patient supplied medication' selected)

☐ Note routed to **DPH AMB WPIC ETE POOL**
☐ Patient to return after medication delivered to MXM (every Thursday)

.LAARTREFERRAL

WPIC Injectable ART Referral - any medical provider may complete and forward to HIV Clinical Leads

WPIC LA-ART REFERRAL

ELIGIBILITY SCREENING

☐ Engaged with WPIC ≥3 months (at least monthly contact)?
☐ Does not plan to leave SF within next 12 months?
☐ CD4 <200 and/or demonstrated difficulty with oral ART adherence ≥1 year?
☐ Has reliable method of contact for injection reminders?
☐ Agrees to monthly injections + lab draws until suppressed, then labs q3 months?

PSYCHOSOCIAL & MEDICAL CONSIDERATIONS

- Substance use disorders? {Yes-describe/No:25117}
- History of psychosis? {Yes-describe/No:25117}
- Taking contraindicated medications (anticonvulsants, rifamycins, chronic systemic glucocorticoids, St. John's Wort)? {Yes-describe/No:25117}

CONTACT INFORMATION

- Location generally sleeps: {housing:19197::"@STREET@","unsheltered - ***","***"}
- Phone/email: {contact:19197::"@PHONE@","@EMAIL@","none","***"}
- Best way to contact for injection reminders: ***
- Community Contacts {friends, partners, case managers, etc:30373}

Name	Relationship	Phone/Email	OK for us to contact if late for injection?
***	***	***	☐ Yes ☐ No
***	***	***	☐ Yes ☐ No {'Ctrl +]' to add row:30373}

{Route this note to DPH AMB WPIC ETE POOL and WPIC HIV Clinical Leads (Nicky Mehtani, MD & Stephen Matzat, MD). Patient should present to MXM for Monday morning clinic.:30373}

.LAARTSTART

WPIC Injectable ART Initiation for HIV Treatment - HIV Clinical Leads only

REVIEW REFERRAL

☐ .LAARTREFERRAL completed?
☐ Contact info and co-morbidities confirmed

RELEVANT LABS

@WPICHIVLABRESULTS@

HIV TREATMENT HISTORY

- Year of Diagnosis: ***
- Risk factors for HIV: ***
- Current ARV regimen: ***
- Prior ARV regimens: ***
- Primary reason(s) for interest in LA-ART: ***

RESISTANCE REVIEW

CAB/RPV clinically significant mutations, for reference:

RPV	*L100I, K101E/P/Q, E138A/G/K/Q/R, Y181C/I, Y188L, H221Y, P225H, F227C, M230L*
CAB	*T97A, G118R, Q148H/K/R/S, E138A/K, G140A/R/S, N155H, R263K*

Known mutations, from all genotypes:

- INSTI mutations: ***
- NNRTI mutations: ***
- NRTI mutations: ***
- PI mutations: ***

☐ {resistance:19197::"No known/suspected resistance to CAB/RPV","Some resistance suspected, ***"}

COUNSELING

☐ Patient verbalizes understanding:
- LA-ART is a long-term/permanent plan; if late → high resistance risk to INSTI + NNRTI classes
- If discontinuing → must take daily oral ART for ≥12 months
- ISRs are common but improve after first dose

☐ Patient agrees if >7 days late for injection:
- Restart previous oral ART (e.g., DRV/c/TAF/FTC) until next injection
- Allow ETE team to contact emergency contacts and conduct mobile outreach

ASSESSMENT & PLAN

Patient confirmed as appropriate candidate for LA-ART.
- Rx plan: ***
- 1st loading dose after medication delivery this upcoming Thursday.

ORDERING CHECKLIST

☐ AMB e-Scripts to AHF Pharmacy (with .AHFNOTE):
- Loading dose: CAB/RPV 600/900mg ×1
- Maintenance doses: CAB/RPV 400/600mg ×3 auto-refills
- LEN if indicated: loading + maintenance doses

☐ CAM orders:
- Loading dose: CAB/RPV 600/900mg PRN "patient presents for 1st dose"
- Maintenance doses: CAB/RPV 400/600mg q28 days

☐ PSA form completed: https://form.jotform.com/231297965730162
☐ Lab orders: HIV VL ×10, CD4 ×3, HIV Genotype ×1 (if viremic), STI labs if sexually active
☐ Chart routed to DPH AMB WPIC ETE POOL

.AHFNOTE

Standardized communication with AHF Pharmacy, place in 'Note to Pharmacy' for e-Rx order.

Deliver to MXM (555 Stevenson) with next weekly delivery. Medi-Cal ID: 000 - (County Indigent) . SSN: xxx-xx-0001 . Meds: *** .
Allergies: is allergic to iodinated contr... .
Select checkbox for "Alternate Address for Delivery" and input MXM
Don't forget to complete AHF's online form.
If SmartPhrase above doesn't fit in order section, send to castropharmacy@ahfrx.org or fax 866-283-4863.

Afterward

About this Protocol

This guide outlines the clinical and operational workflows for the use of long-acting injectable and implantable HIV prevention and treatment therapies, including intramuscular cabotegravir/rilpivirine (CAB/RPV), intramuscular cabotegravir (CAB), and subcutaneous lenacapavir (LEN), for select patients with or at risk for HIV receiving care through Whole Person Integrated Care (WPIC), a division of the San Francisco Department of Public Health. WPIC provides low-barrier healthcare to people experiencing homelessness through its Federally Qualified Health Center, the Maria X Martinez Health Resource Center (MXM), and through community-based delivery models including Street Medicine teams, shelter clinics, and syringe access sites. This decentralized care model supports medication access for a highly mobile patient population.

Several components have been adapted from the "Ward 86 Long-Acting Injectable Antiretroviral Guidelines." Additional considerations reflect experiences specific to the clinical and sociodemographic characteristics of patients experiencing homelessness and accessing care within our low-barrier model. To review the latest Ward 86 guidelines, please see link on the Getting to Zero San Francisco website: https://gettingtozerosf.org/getting-to-zero-resources/

What's New in This Version

- Lenacapavir (Yeztugo) for PrEP: Added as an option with 26-week dosing.
- Traffic Light Tables: Consolidated decision support for timing-based injection administration.
- Smartphrases: Reformatted for usability.
- Counseling Scripts: Added explicit language for "tail" counseling and LEN-specific interactions.
- Structure: Reorganized to improve ease of scanning with a Table of Contents and Quick Reference section.

Questions or Feedback

- Protocol maintained by: WPIC ETE Team
- Last updated: January 2026
