# Supplementary material for: Long-Acting Antiretroviral Therapy for HIV via Drop-in Community-Based Care
Source: JAMA Netw Open. 2026 Jun 26;9(6):e2620348. doi: 10.1001/jamanetworkopen.2026.20348 (PMC13309861; doi:10.1001/jamanetworkopen.2026.20348)
Supplement: Supplement 2. — Data Sharing Statement [file jamanetwopen-e2620348-s002.pdf]

Data Sharing Statement

Mehtani. Long-Acting Antiretroviral Therapy for HIV via Drop-in Community-Based Care. *JAMA Netw Open*. Published June 26, 2026. doi:10.1001/jamanetworkopen.2026.20348

Data
Data available: No

Additional Information
Explanation for why data not available: Because this project was conducted as a quality improvement initiative within a single safety-net clinic, the dataset includes identifiable health information from a small and highly vulnerable population. De-identification could compromise confidentiality, and data sharing was not part of the original institutional approval. Aggregate results are reported in full to ensure transparency.
